# Extraction of Blood Vessels in Retinal Images Using Four Different Techniques

**DOI:** 10.1155/2013/408120

**Published:** 2013-12-17

**Authors:** Asloob Ahmad Mudassar, Saira Butt

**Affiliations:** ^1^Department of Physics and Applied Mathematics, Pakistan Institute of Engineering and Applied Sciences, P.O. Nilore, Islamabad 45650, Pakistan; ^2^Isotope Application Division, Pakistan Institute of Nuclear Science and Technology, P.O. Nilore, Islamabad 45650, Pakistan

## Abstract

A variety of blood vessel extraction (BVE) techniques exist in the literature, but they do not always lead to acceptable solutions especially in the presence of anomalies where the reported work is limited. Four techniques are presented for BVE: (1) BVE using Image Line Cross-Sections (ILCS), (2) BVE using Edge Enhancement and Edge Detection (EEED), (3) BVE using Modified Matched Filtering (MMF), and (4) BVE using Continuation Algorithm (CA). These four techniques have been designed especially for abnormal retinal images containing low vessel contrasts, drusen, exudates, and other artifacts. The four techniques were applied to 30 abnormal retinal images, and the success rate was found to be (95 to 99%) for CA, (88–91%) for EEED, (80–85%) for MMF, and (74–78%) for ILCS. Application of these four techniques to 105 normal retinal images gave improved results: (99-100%) for CA, (96–98%) for EEED, (94-95%) for MMF, and (88–93%) for ILCS. Investigations revealed that the four techniques in the order of increasing performance could be arranged as ILCS, MMF, EEED, and CA. Here we demonstrate these four techniques for abnormal retinal images only. ILCS, EEED, and CA are novel additions whereas MMF is an improved and modified version of an existing matched filtering technique. CA is a promising technique.

## 1. Introduction

Accurate and automatic assessment of retinal images has been considered as a powerful tool for the diagnosis of retinal disorders such as diabetic retinopathy, hypertension, and arteriosclerosis. Blood vessels have varying contrast due to which the darker vessels (thick vessels) can be extracted easily using standard techniques mentioned in the literature while it is difficult to extract the vessels having poor contrast (thin vessels). Segmentation of blood vessels in retinal images is a field of interest for scientists since last two decades [1–4]. Various kinds of eye abnormalities are indicated by changes in vessel tree structure [5, 6]. A true vessel tree structure should contain information about precise thickness of blood vessels in the retinal images. Optic disc and fovea can be located by tracking the vessel tree [7]. Central retinal artery occlusion produces dilated tortuous veins, age related macular degeneration and diabetes can generate new blood vessels (neovascularization), and the study of retinopathy of prematurity in premature infants is not possible without the knowledge of vessel tree structure. Progression of such eye diseases can only be tracked by noticing the changes in the vessel tree structure with the passage of time. Techniques mentioned in the literature [1–32] work well for normal retinal images. Normal images are those containing high contrast vessels and uniform background illumination and which do not contain eye abnormalities such as drusen, exudates, lesions, and microaneurysms. Extraction of vessel tree for normal images is not much useful in comparison with the abnormal retinal images which convey useful information about the progression of different eye abnormalities.

A retinal image has blood vessels with varying thicknesses (36 micron to 180 micron) and varying foreground illumination. The contrast of the blood vessels also varies: higher for thick vessels and lower for thin vessels. In the presence of anomalies in the retinal images, extraction of tree structure becomes more difficult. A variety of vessel tree extraction methods exist in the literature [1–32]. Some of them are kernel based methods such as edge detection filters [8] and matched filters [9, 10]. In matched filter methods if a larger kernel is selected, thick vessels are obtained precisely whereas thin vessels with increased thickness are obtained. The use of smaller kernels can help to precisely select the thin vessels with higher correlation, but the thick vessels are obtained with reduced thicknesses. A conventional matched filtering technique thus requires a large number of different sized kernels with different orientations. Different images corresponding to different kernels are combined to obtain the final tree structure. Local and region based properties to segment blood vessels have been reported in [1] using a probing technique. Pixels are classified as vessels or nonvessels by iteratively decreasing threshold. An automated tortuosity measurement technique for tree extraction is reported in [5]. The method in [5] uses matched filtering, thresholding, thinning and linear classifier algorithm to obtain vessel tree. A classification rate of 91% of blood vessel segmentation and 95% of the vessel network was reported. Some relatively new vessel segmentation techniques which work well for normal images have been reported in [21–31]. The readers may be interested in recent work on localisation and segmentation of optic disc in retinal images [33, 34].

**Figure 1:**
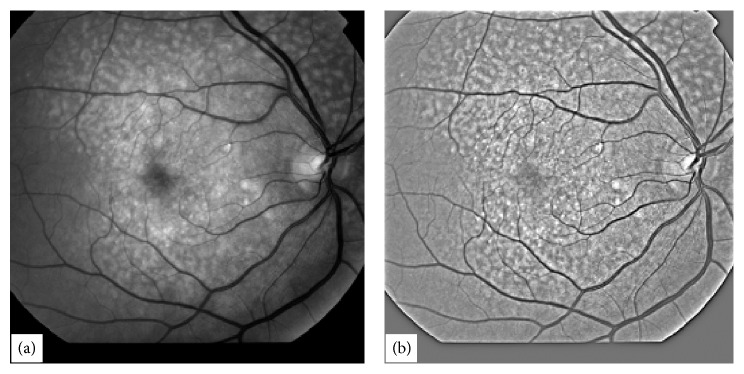
A typical abnormal retinal image in (a) with its homomorphic-processed image in (b).

In this paper, we are presenting four vessel tree extraction techniques. The first one processes the given retinal image by extracting horizontal line cross-sections which are thresholded according to local statistical properties of the line data and the binary lines are combined to obtain binary vessel tree. We are referring this technique as Binary Vessel Extraction (BVE) using Image Line Cross-Sections (ILCS). We are referring the second BVE technique as Edge Enhancement and Edge Detection (EEED). In this technique a minimum of the two images: original retinal image and its blurred version, is generated which is further blurred by using a LOG filter which gives an enhanced version of blood vessels. The image is thresholded using OTSU and the noise is removed from the binary image. The resulting image gives a free of noise blood vessel tree. The third technique that we are reporting is the Modified Matched Filtering (MMF) technique. In MMF preprocessing of the original retinal image includes application of homomorphic filtering for contrast enhancement, assignment of thresholded value (obtained using OTSU) to all image pixels with threshold value while retaining lower image values for enhancement of blood vessels. The image is then subjected to conventional matched filtering and is thresholded by OTSU for vessel tree extraction. The fourth technique is based on an algorithm which we are referring to as Continuation Algorithm. In CA two binary images: one containing thick vessels (reference image) and the other containing thin vessels plus the thick vessels (test image), are processed. The vessel tree in the reference image is extended with the help of vessel tree in the test image. When further extension is halted, the reference image contains a complete blood vessel tree. The images presented in this paper were either taken from STARE [11] or from Alexandra Eye Pavilion Hospital, Edinburgh, UK. From the available images only 30 images were chosen for BVE by these four methods which were abnormal and were hard to process with the techniques given in the references [1–31]. Out of the thirty blurred retinal images, we are reporting the images with highest abnormalities. One image as shown in Figure 1(a) was processed with all the four techniques for intercomparison. The three techniques ILCS, EEED, and CA are novel and the fourth technique MMF is an improved and modified version of the existing technique called matched filtering. Matched filtering technique is one of the most cited techniques in literature and has been included to make comparison with the rest of the three techniques. We are describing these four methods for BVE one by one in the following sections. In this paper, our main emphasis is on the extraction of blood vessels from abnormal retinal images. We have also tested these four techniques on 105 normal retinal images with higher success rates, but we are not reporting them as they form trivial cases which can be dealt successfully with most of the techniques given in the references [1–32].

## 2. Blood Vessel Extraction (BVE) with Image Line Cross-Section (ILCS)

**Figure 2:**
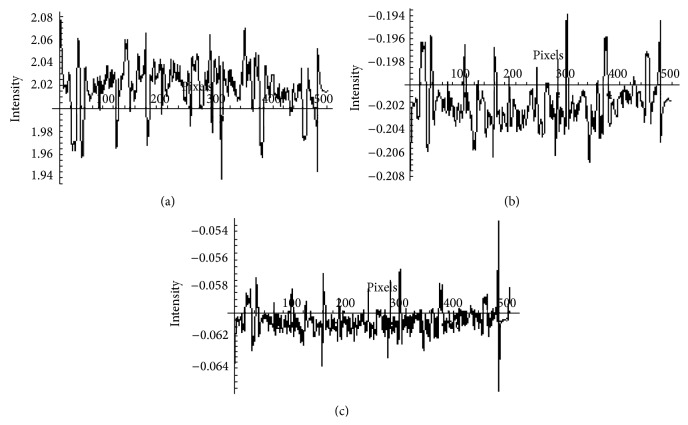
A typical cross-section of a retinal image and its processing, (a) a typical horizontal cross-section of the image in Figure 1(b), (b) convolution of the pattern in (a) with kernel1, (c) convolution of the pattern in (b) with kernel2.

Retinal images have inhomogeneous illumination pattern due to built-in shape of retina. To remove inhomogeneity in illumination [12] the retinal images are processed first with homomorphic filtering [8]. A typical poor quality highly abnormal retinal image is shown in Figure 1(a). The image in Figure 1(a) was processed with homomorphic filter which is shown in Figure 1(b). Horizontal cross-sections of the image in Figure 1(b) are extracted one by one for further processing. A typical horizontal cross-section from the image in Figure 1(b) is shown in Figure 2(a).

The data shown in Figure 2(a) is convolved with kern1 = {0, − 0.1, 0}, and the resulting data is shown in Figure 2(b). The application of the convolution process with kern1 has resulted in the inversion of the data. The data shown in Figure 2(b) is then convolved with kern2 = {−0.35, 1, − 0.35} and the resulting data is shown in Figure 2(c). The application of the convolution process with kern2 has produced vessels with improved signal to noise ratio. The horizontal axis shown on the plot of Figure 2(c) corresponds to the threshold level given by (1). The kern1 and kern2 were determined on the basis of a set of experiments yielding best results in terms of signal to noise ratio for blood vessels

(1) $$\begin{array}{c} \text{threshold}=\bar{x}+\alpha \cdot \frac{\text{standard}\;\;\text{deviation}}{\text{rm}{\text{s}}^{2}}, \end{array}$$

where

(2) $$\begin{array}{c} \text{standard}\;\;\text{deviation}=\sqrt{\frac{\sum_{i}{\left(x_{i}-\overset{-}{x}\right)}^{2}}{n-1},}\\ \text{rms}=\sqrt{\frac{\sum_{i}x_{i}^{2}}{n-1},} \end{array}$$

**Figure 3:**
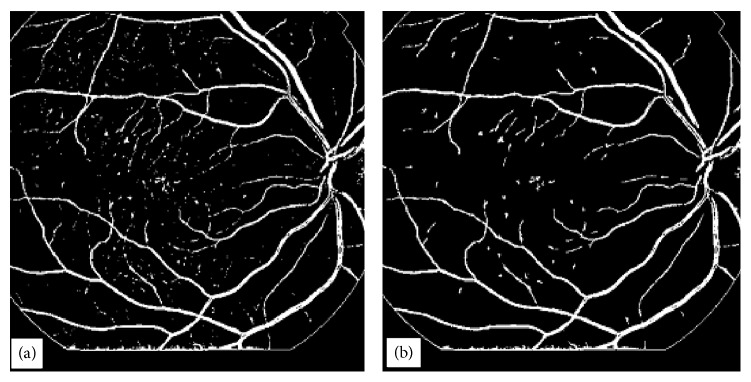
BVE with ILCS, (a) vessel tree extracted from image in Figure 1(b) with processing of image line cross sections, (b) image obtained from (a) after length filtering at 10 pixels.

*n* is the of number data points in a given line cross section, *x*_*i*_ represents the *i*th data point, and $\overset{-}{x}$ represents the mean value of the data in a line cross section. *α* is a constant equal to a value of 3.3 × 10^−3^, and this value has been found to give very satisfactory threshold values for all the cross sections in 96% retinal images tested. Some retinal images with very poor contrast required a small variation in the value of *α* to give an excellent tree structure. Once all the line cross sections of a retinal image are processed, they are combined to form a 2D binary vessel tree as shown in Figure 3(a).

This method has been implemented on 30 highly abnormal retinal images, and 78% efficiency was achieved for high contrast thick blood vessels and for low contrast blood vessels the efficiency varied from 74 to 78% depending upon the contrast of the blood vessels against background. Thin blood vessels with contrast as small as 0.01 were extracted with 77% efficiency using the proposed method. However, blood vessels contrast can be further improved by using histogram equalization method in overlapping windows with unit step and then applying line cross-section method on this contrast enhanced image. The image in Figure 3(a) after length filtering at 10 pixels is shown in Figure 3(b).

**Figure 4:**
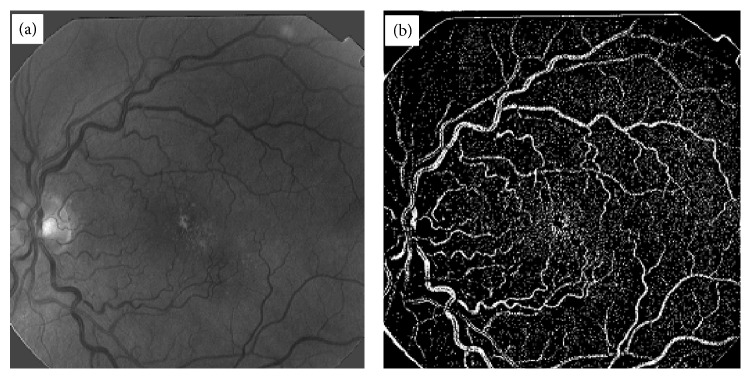
Demonstration of ILCS technique for a noisy retinal image, (a) a typical abnormal and noisy retinal image, (b) vessel tree image obtained from the image in (a) using ILCS method.

**Figure 5:**
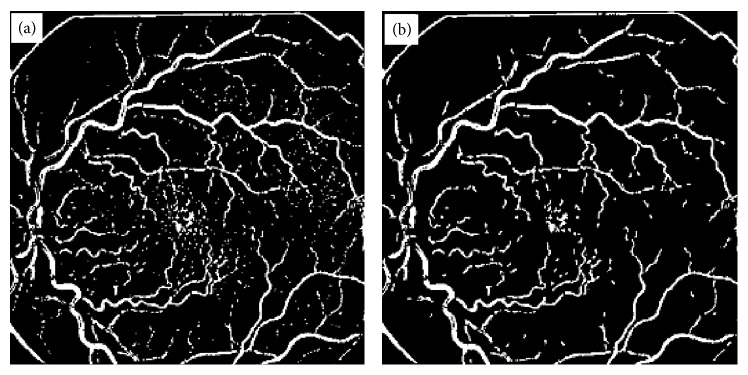
Demonstration of ILCS technique for a noisy retinal image blurred with Gaussian filter, (a) vessel tree obtained from the image in Figure 4(a) after application of Gaussian blurring filter, (b) vessel tree image in part (a) after length filtering at 10 pixels.

Length filtering is an algorithm by which small isolated structures most probably nonvessels are removed from the image. The original thickness of all the blood vessels has been exactly reproduced without any artifacts by ILCS. Widths of vessels were found to be preserved by the ILCS method. Some retinal images contain a lot of noise and when such images are processed for BVE using ILCS the resulting vessel tree also contains a lot of noise. An example of such a retinal image is shown in Figure 4(a), and vessel tree extracted by ILCS is shown in Figure 4(b). The thin vessels in the image shown in Figure 4(a) have very poor contrast which matches with noise spread over the image background. The resulting vessel tree thus contains enhancement of background noise also. An application of length filtering technique at this stage for noise removal also removes a significant portion of thin blood vessels. To make ILCS more effective, we suggest a slight blurring of such retinal images with a small sized Gaussian kernel of standard deviation *σ* = 0.824 and then suggest the application of ILCS on resulting blurred retinal images. A vessel tree obtained in this way is shown in Figure 5(a) and after application of length filtering at 10 pixels is shown in Figure 5(b).

**Figure 6:**
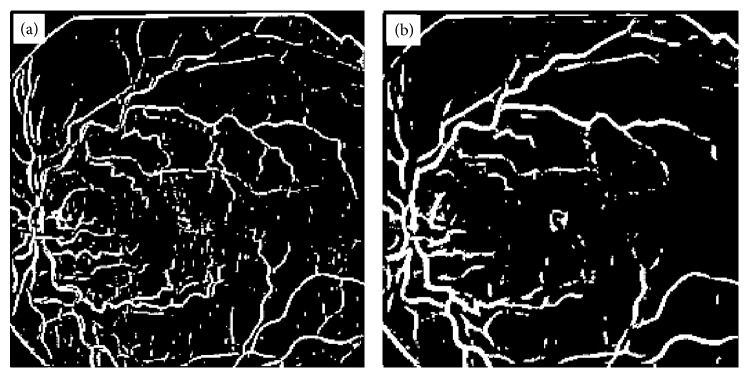
Vessel tree results obtained with conventional matched filtering technique [9], (a) vessel tree obtained using matched filtering kernel of length 2 and width 4, (b) vessel tree obtained using matched filtering kernel of length 4 and width 6.

**Figure 7:**
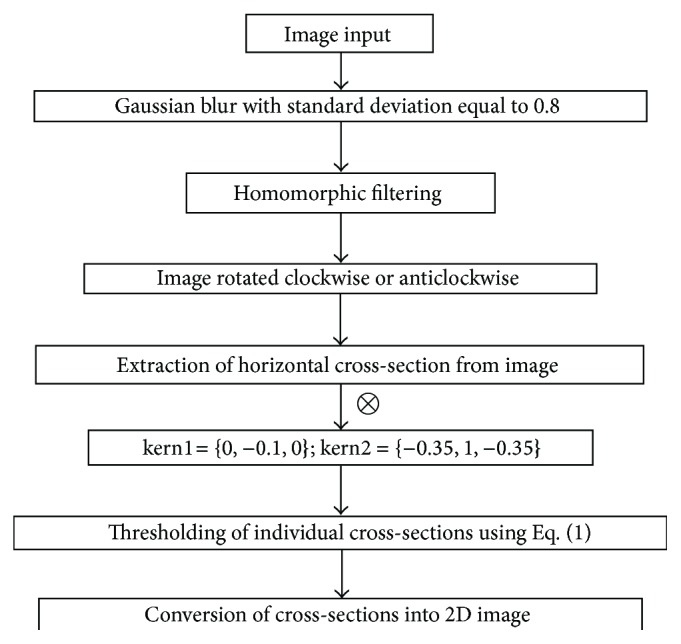
Flow chart of the proposed vessel segmentation technique based on line cross-sections.

Figure 6 shows the results obtained with two different matched filters (of different lengths and widths) when applied to the image shown in Figure 4(a). The vessel trees obtained with matched filtering technique shown in Figure 6 can be compared with the result obtained with ILCS shown in Figure 5(b). The result shown in Figure 6(a) corresponds to a matched filter kernel of length 2 and width 4, whereas the result shown in Figure 6(b) corresponds to matched filter of length 4 and width 6. The drawback of the matched filtering method, as can be easily seen from these results, is that the shape of tortuous blood vessels has been greatly exaggerated near the optic disc region in Figure 6(b). The reason for the failure is that each matched filter kernel is designed for a specific environment related to the thickness and linearity of the vessels. Since in normal retinal images blood vessels are not very tortuous, therefore matched filtering can work well, but in case of retinal images with tortuous vessels (an indication of hypertension), it has not worked due to mismatching of filter parameters with the nature of vessels. So in general terms we can conclude that the matched filtering technique is not appropriate option especially when the images are abnormal containing drusen or tortuous vessels. The working principle of the proposed technique has been explained through the flow chart diagram given in Figure 7. Some important vessel segmentation techniques reported so far in the literature [1–31] are based on some sort of thresholding criterion. The techniques either use threshold selection based on the whole image data (called global thresholding) or data from the patches of the image (called adaptive thresholding). The thresholding in ILCS is based on the horizontal line cross-section of the image under process and the selection of threshold is based on (1). The way the ILCS extracts vessel tree from retinal images is entirely a new concept.

## 3. Blood Vessel Extraction (BVE) Using Edge Enhancement and Edge Detection (EEED)

**Figure 8:**
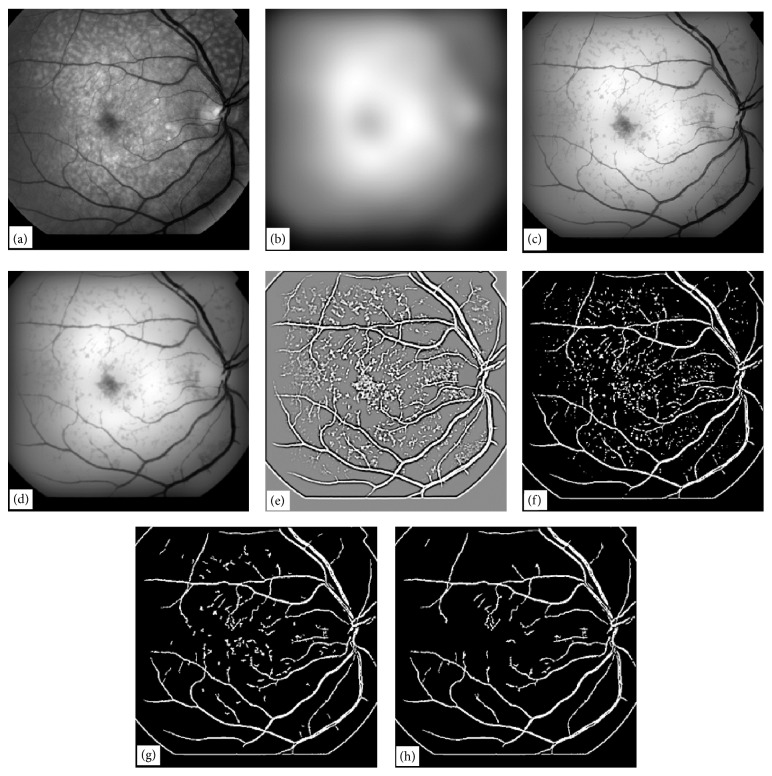
Illustration of a vessel extraction technique by EEED, (a) original retinal image, (b) Gaussian blur of the image in (a) with *σ* = 24, (c) minimum of the images in (a) and (b), (d) Gaussian blur of the image in (c) width *σ* = 1, (e) result of LOG filter convolution of size (9, 9) with image in (d), (f) optimum threshold of the image in (e), (g) application of noise deletion filter of window size (8, 8) to image in (f), (h) application of noise deletion filter of window size (16, 16) to image in (g).

In this section, we address the issue of extraction of blood vessels in retinal images using a novel edge enhancement and edge detection technique. The proposed method also clears the unwanted edges which are not blood vessels. This technique is very robust and fast. Most of the techniques mentioned in [1–31] work well for normal retinal images containing no abnormalities. As the retinal image becomes abnormal due to the inclusion of drusen or due to lower image contrast, the performance of the techniques in [1–31] falls and the vessel tree structures obtained with those techniques do not represent the actual blood vessels tree. To demonstrate the capability of EEED we have chosen an image shown in Figure 8(a) which is low in contrast and at the same time contains a large number of drusen spread over the entire retinal image. The techniques mentioned in [1–31] do not give satisfactory results particularly for images like the one shown in Figure 8(a). The main objectives of the EEED technique are to enhance the contrast of blood vessels and at the same time diffuse the other abnormal features present in the retinal image. These objectives need to be achieved in a single go. Application of Laplacian of Gaussian (LOG) filter further enhances the vessel contrast and suppresses the other abnormal image features. The image can then simply be thresholded using any standard technique, for example, well known OTSU thresholding technique can be applied to obtain binary vessel tree.

For the extraction of blood vessels with EEED, a retinal image is first convolved with a large Gaussian blurring kernel. The blurred image will lose all the details contained in the retinal image and will contain only the illumination pattern. Once a Gaussian blurred image is obtained from a retinal image, another image is formed out of the two images. The resulting image is actually a minimum of the two images. The blood vessels in a retinal image normally have intensities lying in the lower range. The minimum image enhances this fact; that is, the blood vessels have the lowest values with enhanced edges in comparison with background. The background in the minimum image is completely diffused suppressing information about the drusen and the optic disc.

The minimum image is then blurred a little bit, normally with a Gaussian blurring technique with a small kernel. This process helps to develop continuity in the broken pieces of vessel tree structure. The blurred-minimum image is then convolved with a LOG filter with a kernel size of (9, 9). The resultant image is contrast enhanced and contrast reversed with more prominent vessel trees and more uniform background. If the retinal image contains noise comparable with the contrast of vessel trees, then the noise will also be enhanced. Due to contrast reversal, the vessels now look bright with boundaries having dark edges. This is a typical feature which appears with the use of LOG filters.

The application of the LOG filter gives an image with uniform background intensity. This feature is similar to the use of a homomorphic filter on a retinal image. Due to uniform background intensity and the vessel tree having higher intensities as compared with background, the image is simply thresholded using any optimum thresholding technique. We used the OTSU algorithm to obtain optimum thresholding for the image obtained with the LOG filter. The thresholding process converts the LOG filtered image into a binary image. The binary image consists of the blood vessel tree structure and noise. Some images at this stage have more noise and some have relatively less.

Noise in the binary images can be eliminated using a length filtering technique or a noise removal technique. We developed our own noise removal algorithm for binary images. The algorithm works in windows of sizes (*x*, *x*) where *x* may vary from 4 to 16. In this algorithm all the pixel values on the boundary of a window are summed up. If all the boundary pixels have zero values, that is, the sum of boundary pixel values is zero, then all the pixels within the window are deleted. The working of this algorithm gave satisfactory results. After application of this algorithm the image obtained contained only the vessel tree structure. This method is fast and does not involve human intervention at any stage.

To illustrate the EEED technique for vessel tree extraction, we chose a retinal image as shown in Figure 8(a). This image was convolved with a Gaussian blurring kernel of *σ* = 24. The blurred image so obtained is shown in Figure 8(b). The minimum of the two images, (image in Figure 8(a) and image in Figure 8(b)) was computed and the resultant image is shown in Figure 8(c). The image in Figure 8(c) is a vessel enhanced image on a nonuniform background. The image in Figure 8(c) was then convolved with a Gaussian blurring kernel of *σ* = 1. This process helps to develop continuity in the vessel tree structure; otherwise broken or missing pixels in the binary vessel tree will be observed. The image obtained in this process is shown in Figure 8(d).

An optimum threshold computed on the image in Figure 8(d) does not give an appropriate vessel tree because the illumination of the background is still nonuniform. To eliminate this nonuniform background problem, we convolved the image in Figure 8(d) with a LOG kernel of size (9, 9). The resulting image is shown in Figure 8(e). This image is contrast reversed in which the blood vessels have brighter intensities. The bright vessels have dark edges as well. This is a typical feature of the LOG filter convolution process. The background is uniform in the image in Figure 8(e). An optimum threshold was computed for the image in Figure 8(e) and the binary image so obtained is shown in Figure 8(f). The image in Figure 8(f) has binary noise which is not a part of the vessel tree structure. The noise in this image is undesired.

The noise in the image shown in Figure 8(f) can be removed using a number of techniques. We applied the length-filtering technique to remove the binary noise which is either not a part of vessel tree or a small broken isolated part of a vessel tree. Length filtering algorithm has been explained earlier. A window of size (8, 8) was chosen and the noise removal algorithm was applied to the image in Figure 8(f). The resultant image is shown in Figure 8(g). The image in Figure 8(g) has much of the noise removed, but there is some noise which can still be removed. To remove the rest of the noise in image in Figure 8(g) we applied the noise removal algorithm with a window size of (16, 16) and obtained a completely noise free image, which is shown in Figure 8(h). The image in Figure 8(h) is the final step in our novel EEED technique for vessel tree extraction. The novel technique for vessel extraction presented in this section has been tested on 30 retinal images of varying illumination and contrast, and the success rate without human intervention was (88-89%) and with human intervention was (90 to 91%). One of the 30 images required human intervention for filtering the image to produce the best result. We have found that EEED technique gave excellent results. For normal retinal images, EEED gave improved results and the success rate varied in the range (96 to 98%) when tested on 105 retinal images.

## 4. Blood Vessel Extraction (BVE) Using Modified Matched Filtering (MMF)

In this section matched filtering method for extraction of blood vessels is used with preprocessing in order to get improved quality of the extracted blood vessels. The important feature in our method is that it produces good quality totally automatic BVE, which can be useful for the eye care professionals for patients screening, treatment, evaluation, and clinical study. In this approach the image background at corners is first modified and then a homomorphic filter is applied to smooth the images. This will enhance the contrast of the images in comparison with the original images. Since we are interested in enhancing the signal of the blood vessels which lie in the range below the threshold value, the image is automatically thresholded using the OTSU algorithm keeping the information in the image below the threshold value and assigning the rest of the image the same value as the threshold. This will greatly enhance the contrast of the blood vessels. We have done this because our required data lies towards the lower end of the image data spectrum. This act has greatly enhanced the contrast of the image. Then this image is subjected to the matched filtering technique and thresholded automatically by OTSU algorithm. 80 to 85% was the success rate in obtaining correct vessel trees by MMF without human intervention. An improvement of nearly 2% occurs if human intervention is involved. This is because by assigning threshold value to higher intensity spectrum leads to a decrease in within-class variance and an increase in between-class variance. Then matched filtering technique is applied to that enhanced image and resultant image is thresholded automatically. This method is fast and totally automatic. The work presented in this section is more relevant to the work reported in [1, 10, 18, 19].

A matched filter technique has been presented for the purpose of enhancement of blood vessels in retinal images [19]. This technique is fully automated for thresholding purposes in order to get a binary tree of blood vessels. The method used for automatic threshold selection is known as the OTSU method of threshold determination [18]. Necessary modifications have been suggested in order to achieve better results. The physical concept behind this method is summarized as follows.

Three properties of the blood vessels in retinal images may be noted. In a 2D retinal image, blood vessels may have three possible orientations: one orientation is when the blood vessels are along the horizontal axis of the image (say *x*-axis), blood vessels may be oriented along the vertical axis (say *y*-axis), or the blood vessels may be at some angle with the *x*-axis. The blood vessels at some angle *θ* are approximated by piecewise linear segments. Blood vessels can have small curvatures with antiparallel curvature edges and these anti-parallel pairs may also be approximated by piecewise linear segments.

Blood vessels are darker relative to the surrounding region in a retinal image due to lower reflectance relative to the surrounding regions. Blood vessels seem to follow an inverted Gaussian profile with the width of the vessels varying from 2 pixels to 10 pixels for a standard image. This range may correspond to 36 to 180 microns. To introduce the concept of matched filtering in retinal images, let us consider (3) which is used in the theory of communication [22, 32]

(3) $$\begin{array}{c} s_{o}\left(t\right)=\int H\left(f\right)\left\{S\left(f\right)+\eta \left(f\right)\right\}\cdot e^{i\cdot 2\pi \cdot f\cdot t}df, \end{array}$$

where *s*_*o*_(*t*) is the output, *s*(*t*) is the input with an additive Gaussian noise *n*(*t*), such that *S*(*f*) = *ℑ*{*s*(*t*)} and *η*(*f*) = *ℑ*{*n*(*t*)}, where *ℑ* is the Fourier transform operator and *f* is the variable in the frequency domain corresponding to the variable *t* in the time domain. *H*(*f*) = *ℑ*{*h*(*t*)} is the transfer function of the system with impulse response *h*(*t*). It has been shown [10] that the filter *H*(*f*) that maximizes the signal to noise ratio for *s*_*o*_(*t*) is given by *H*_opt_(*f*) = *ℑ*{*s*(−*t*)} = *S* ^*^(*f*), where *H*_opt_(*f*) is known as the matched filter with impulse response *h*_opt_(*t*) = *s*(−*t*) = *s*(*t*), when *s*(*t*) is an even function. In a typical communication system, if there are *n* different inputs *s*_*i*_(*t*), *i* = 1,2,…, *n* received, then these inputs are passed through a stack of *n* matched filters and the output with the maximum value is selected.

For the 2D retinal images, *s*(*t*) needs to be defined at the vessels and may be approximated by an inverted Gaussian function of appropriate width with Gaussian centred at the centre of the vessels. As the vessels move away from the optic disc the width of the vessels narrows down. For the inverted Gaussian to act as a perfect matched filter for the vessels, the width of the Gaussian function should also be narrowed down. As the vessels change their orientation the Gaussian function needs to be rotated as well. We have suggested an inverted Gaussian to act as a matched filter for the enhancement of blood vessels in retinal images. Ideally the matched filter must have all possible orientations and varying thickness to enhance vessels with different orientations and thicknesses. Assuming blood vessels are the signal to be enhanced, *s*(*t*) may take the form given by

(4) $$\begin{array}{c} f\left(x,y\right)=A\left\{1-k\cdot \exp\left(\frac{-d^{2}}{2\sigma^{2}}\right)\right\}, \end{array}$$

where *d* is the perpendicular distance between the point (*x*, *y*) on the vessel and the straight line passing through the centre of the blood vessel in a direction along its length. *σ* corresponds to the spread of the Gaussian intensity profile of the vessel, *A* is the grey-level intensity of the local background, and *k* is a measure of the reflectance of the blood vessel relative to its neighbourhood. The optimal filter to act as a matched filter for the vessels may be written as given by.

(5) $$\begin{array}{c} h\left(d\right)=-\exp\left(\frac{-d^{2}}{2\sigma^{2}}\right). \end{array}$$

The negative sign implies that the vessels are darker than the background. For simulation purposes, (5) for the matched filter in its 2D form can be written as given by.

(6) $$\begin{array}{c} F\left(x,y\right)=-\exp\left(\frac{-x^{2}}{2\sigma^{2}}\right)\;\left|y\right|\leq \frac{L}{2}. \end{array}$$

This will act as an ideal matched filter for a segment of the vessels lying along the *y*-axis and *L* is the length of the vessel segment for which the vessel is assumed to have a fixed orientation. The matched filter above is valid only for a segment of the vessels of certain Gaussian width. This filter must have different values of *σ* to act as a matched filter for different widths of the vessels lying along the *y*-axis. For vessels other than along the *y*-axis, the filter above needs to be rotated to act as a matched filter for vessels with different orientations. In our simulation, we used only one value of *σ* but rotated the filter from 0° to 180° with a step size of 9°. The application of matched filtering with these orientations generates 21 images. Each shows vessel enhancement for a particular orientation of the filter. These images are then combined retaining the maximum value of the pixels among the 21 images. The resultant image gives a vessels enhanced version of the retinal image.

It has been observed that by changing the width of the matched filter, there is a considerable effect on the thickness of the extracted blood vessels. If the filter is very wide, then the blood vessels extracted will be thicker in size as compared with their original width in the image and vice versa. Kernel with width ranging from *w* = 6 to *w* = 8 is the most accurate range for vessel extraction in comparison with the original thickness of the blood vessels.

**Figure 9:**
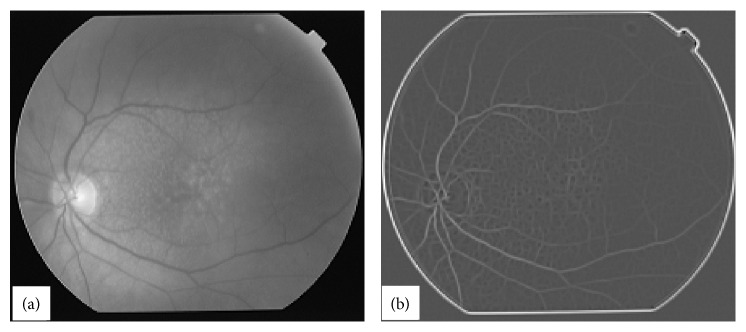
Response of matched filtering on a typical retinal image, (a) retinal image, (b) maximum matched filter response of image in (a).

**Figure 10:**
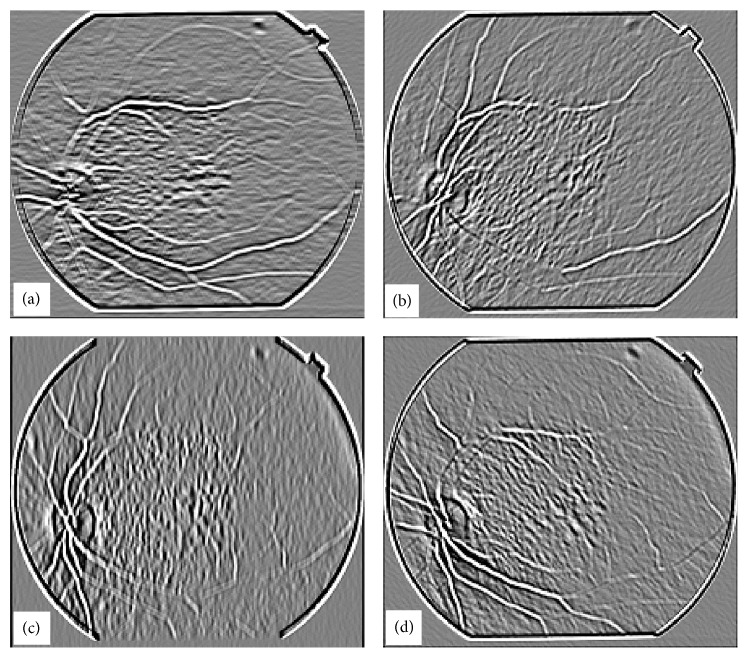
Matched filter results when kernel with *w* = 6 is applied to the image in Figure 1(a) for four different orientations, (a) image obtained when matched filter kernel was at an angle *θ* = 0°, (b) image obtained when matched filter kernel was at an angle *θ* = 45°, (c) image obtained when matched filter kernel was at an angle *θ* = 90°, (d) image obtained when matched filter kernel was at an angle *θ* = 135°.

Figure 9(a) shows an image for blood vessel extraction. This image was processed with a matched filter at *w* = 6 and the response of the matched filter is shown in Figure 9(b).  Figure 10 shows the intermediate stage in which the matched filter response for *w* = 6 is shown at angles of 0°, 45°, 90°, and 135° for the image shown in Figure 9(a). It shows how blood vessels at different locations in an image are enhanced with the rotation of the matched filter kernel.

**Figure 11:**
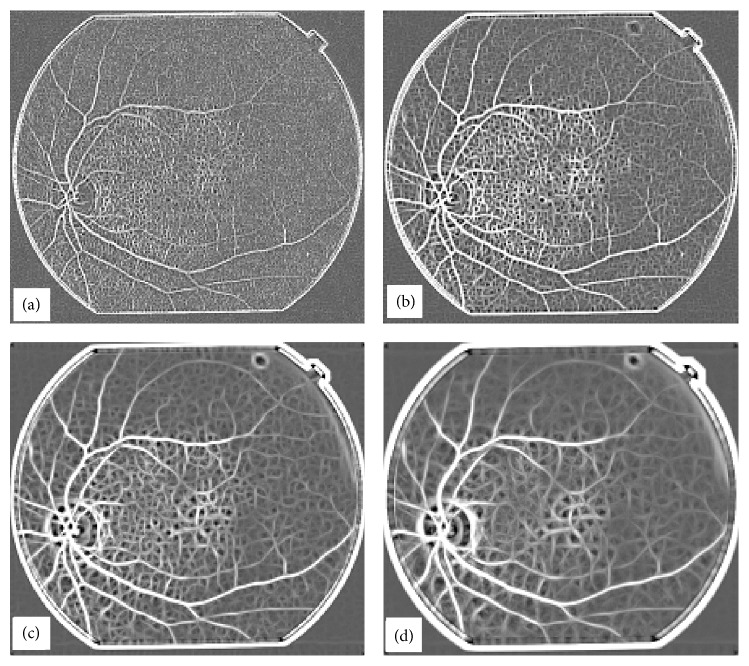
Effect of change in width (*w*) of the matched kernel on retrieved images, (a) *w* = 2, (b) *w* = 4, (c) *w* = 10, (d) *w* = 12.

The results described in Figure 9 are for the suitable range of width of matched filter kernel, but if we increase or decrease the width of the matched filter kernel, then the results obtained are not as good as in the previous case. The width of the Gaussian is determined by *σ* and the sampling is determined by *w*. Appropriate values of both *σ* and *w* are required to obtain optimum matched filter results. The overall response for the matched filter at *w* = 2, 4, 10, 12 is shown in Figure 11.

**Figure 12:**
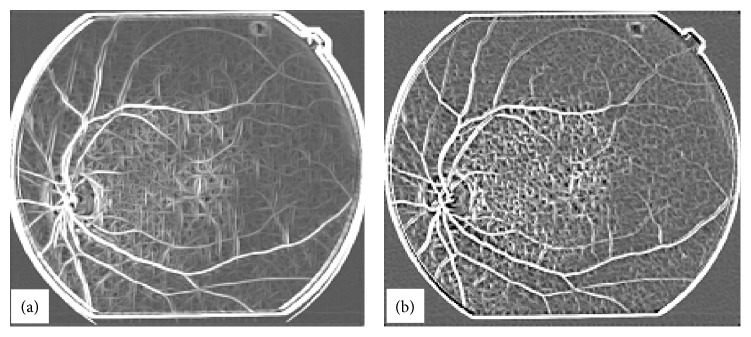
Matched filter response at different values of length of kernel, (a) length of kernel = 2*w*, (b) length of kernel = 0.5*w*.

It was also found that by changing the length of the kernel, there was an adverse effect on the quality of image obtained after the matched filtering technique.  Figure 12(a) is the maximum matched filter response with *w* = 6, but this time length along the *y*-axis has been changed from (−*w*, + *w*) to (−2*w*, + 2*w*); that is, the length of kernel has been doubled while the length along the *x*-axis remained the same as (−*w*, + *w*).  Figure 12(b) shows the result when the length along the *y*-axis has been halved from (−*w*, + *w*) to (−0.5*w*, + 0.5*w*).

In order to get the coordinates of the blood vessels we need to threshold the image obtained after the matched filtering. The selection of an adequate threshold of the grey-level for extracting objects of interest from background is very important. Most of the methods used to threshold the image automatically were based on the image histogram. In the ideal case the histogram for the image should be bimodal. The two peaks represent the background and the foreground. Ideally there is a deep and sharp valley between these two peaks, but practically, for most grey-level images, it is difficult to detect the exact valley bottom precisely, especially when two peaks are unequal in height producing no trace of a valley. If the image histogram is unimodal, such methods do not work. The algorithm used here for automatic thresholding is called OTSU [18]. This method is based on discriminant analysis. In this method threshold operation is regarded as the partitioning of the pixels of an image into two classes.

Let there be *L* grey-levels in a retinal image [1,2,…, *L*]. Let *n*_*i*_ be the number of pixels at level *i*. The total number of pixels in the image is then given by *N* = *n*_1_ + *n*_2_ + ·+*n*_*L*_. The probability of the *i*th grey-level is given by *p*_*i*_ = *n*_*i*_/*N*, where ∑_*i*=1_^*L*^ *p*_*i*_ = 1. Let us assume that we want to divide the grey-levels in the retinal image into two classes C1 and C2 to separate the background from the foreground, where C1 denotes pixels with levels [1,2,…, *k*] and C2 denotes pixels with levels [*k* + 1, *k* + 2,…, *L*], where *k* is the grey-level value that thresholds the background from the foreground. In retinal images information about the blood vessels falls in the category of lower grey-levels. The probability of class occurrence and class mean levels are given, respectively, by the following equations:

(7) $$\begin{array}{c} \omega_{1}=\sum_{i=1}^{k}p_{i},\\ \omega_{2}=\sum_{i=k+1}^{L}p_{i},\\ \mu_{1}=\sum_{i=1}^{k}\frac{i\cdot p_{i}}{\omega_{1}},\\ \mu_{2}=\sum_{i=k+1}^{L}\frac{i\cdot p_{i}}{\omega_{2}}, \end{array}$$

where *ω*_1_, *ω*_2_ is the probability of class occurrences of C1 and C2, respectively, and *μ*_1_,  *μ*_2_ are the class means for C1 and C2, respectively. *μ*_*T*_ = ∑_*i*=1_^*L*^ *i* · *p*_*i*_ is the total mean level of the original image.

It is easy to establish *ω*_1_ · *μ*_1_ + *ω*_2_ · *μ*_2_ = *μ*_*T*_ and *ω*_1_ + *ω*_2_ = 1. The class variances may be found from the following equations:

(8) $$\begin{array}{c} \sigma_{1}^{2}=\sum_{i=1}^{k}\frac{{\left(i-\mu_{1}\right)}^{2}\cdot p_{i}}{\omega_{1}}\\ \sigma_{2}^{2}=\sum_{i=k+1}^{L}\frac{{\left(i-\mu_{2}\right)}^{2}\cdot p_{i}}{\omega_{2}}. \end{array}$$

To determine the “goodness” of the threshold at level *k*, we can use the following discriminant criterion measures or the measures of class separability used in the discriminant analysis [31]:

(9) $$\begin{array}{c} \eta =\frac{\sigma_{B}^{2}}{\sigma_{T}^{2}}, \end{array}$$

where

(10) $$\begin{array}{c} \sigma_{B}^{2}=\sum_{i=1}^{2}\omega_{i}{\left(\mu_{i}-\mu_{T}\right)}^{2}. \end{array}$$

The relationship between *σ*_*B*_^2^ and *σ*_*T*_^2^ is given by

(11) $$\begin{array}{c} \sigma_{B}^{2}+\sigma_{w}^{2}=\sigma_{T}^{2}, \end{array}$$

where *σ*_*w*_^2^ is given by

(12) $$\begin{array}{c} \sigma_{w}^{2}=\omega_{1}\sigma_{1}^{2}+\omega_{2}\sigma_{2}^{2}. \end{array}$$

*σ*_*B*_^2^ and *σ*_*T*_^2^ are the between-class variance and the total variance of levels, respectively, where *σ*_*w*_^2^ is defined as within-class variance. It should be noted that *σ*_*w*_^2^ and *σ*_*B*_^2^ are functions of the threshold *k* where *σ*_*w*_^2^ is based on second order statistics while *σ*_*B*_^2^ is based on the first order statistics and *σ*_*T*_^2^ is independent of *k*. Thus the problem is to find the optimum value of *k* labelled as *k* ^*^ that maximises *η* or equivalently *σ*_*B*_^2^. We can write

(13) $$\begin{array}{c} \sigma_{B}^{2}\left(k^{*}\right)=\underset{1\leq k<L}{\max}\left[\sigma_{B}^{2}\left(k\right)\right]. \end{array}$$

Once the value of *k* ^*^ is determined, the retinal image is split into two levels at the value of *k* ^*^. This method of finding the threshold has also been tested for multilevel thresholding in order to calculate a suitable threshold for the replacement of black regions at corners by a suitable grey-level as shown in Figure 9(a). The black regions at corners are not part of the actual retina. The algorithm worked well by splitting the image into three classes but was unable to classify that darker region into a single separate class.

**Figure 13:**
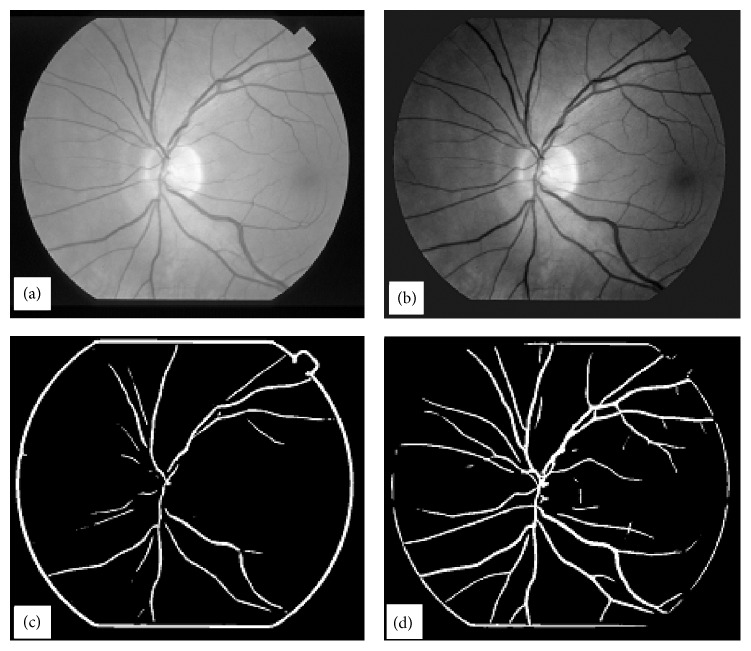
Effect of image background on vessel tree extraction, (a) original Image, (b) background modified image, (c) automatic thresholding of image in (a), (d) automatic thresholding of image in (b).

In order to extract the coordinates of blood vessels in the matched filtered image we have applied the algorithm described earlier also known as OTSU for thresholding. Results have suggested that if we use an image in which the surrounding dark part (corners) is very black, then the threshold selected by the algorithm is not very good. However, if we assign those black regions by a value which is close to image background, then much better results are obtained. The image in Figure 13(a) is modified when the black surrounding corners are replaced with the image mean value. This information is gathered from the image histogram which gives a distinct peak for the background. The modified image is shown in Figure 13(b). Figures 13(c) and 13(d) show the results of automatic thresholding on matched filtered output of the images in Figures 13(a) and 13(b), respectively. If one wants to reveal further detail in the image, for example, if the blood vessels have very poor contrast against the background, the threshold decided by the algorithm can be further reduced manually and one can see the results, but in most cases this will increase the noise in the image.

It has been observed that a better threshold decision takes account of the data of the surrounding corners as well. If the data at these edges is just like the image data, then even better results can be achieved. The reason for better results with modified surroundings can be explained on the basis of (11) which shows that the sum of squares of variances “between the classes” and “within-class” is a constant and is independent of the threshold *k*. In discriminant analysis, within-class variance shows the scatter of samples around their class expected value. Thus by replacing the small value pixels from the surroundings by a higher value the variance within the class will be reduced. In an unmodified image the variance within the class is high while after modification with higher values at these pixels the variance within the class becomes lower. Since the sum of two variances (within-class, between the classes) is constant if the within-class variance is high then the between-class variance will be small and vice versa. Thus by performing the above action of intensity modification at the surrounding pixels we have actually reduced within-class variance and consequently between-class variance increases. From the results of Figure 13(d) we can see that we can extract the coordinates of the main blood vessels in retinal images successfully.

**Figure 14:**
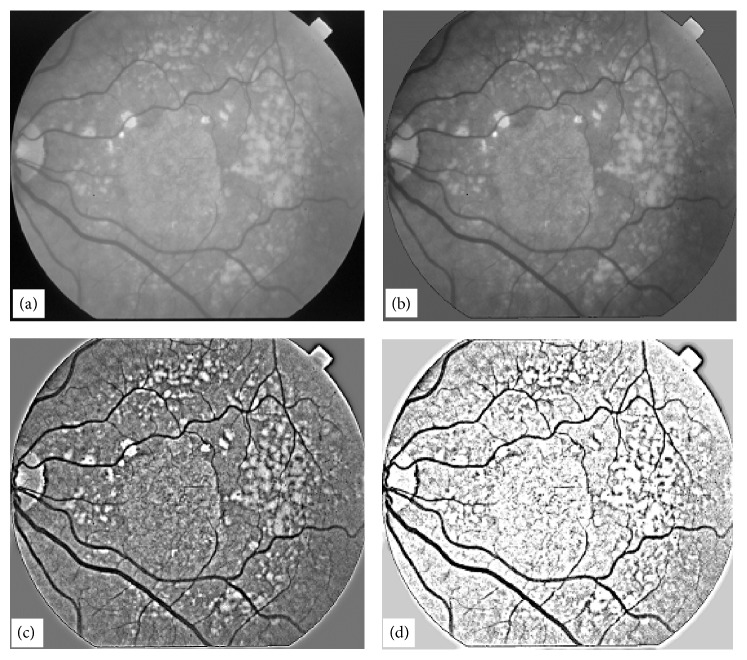
Preprocessing steps for vessel tree extraction, (a) original image, (b) image with modified background of the image in (a), (c) image after homomorphic filtering of image in (b), (d) image after OTSU thresholding on the image in (c).

**Figure 15:**
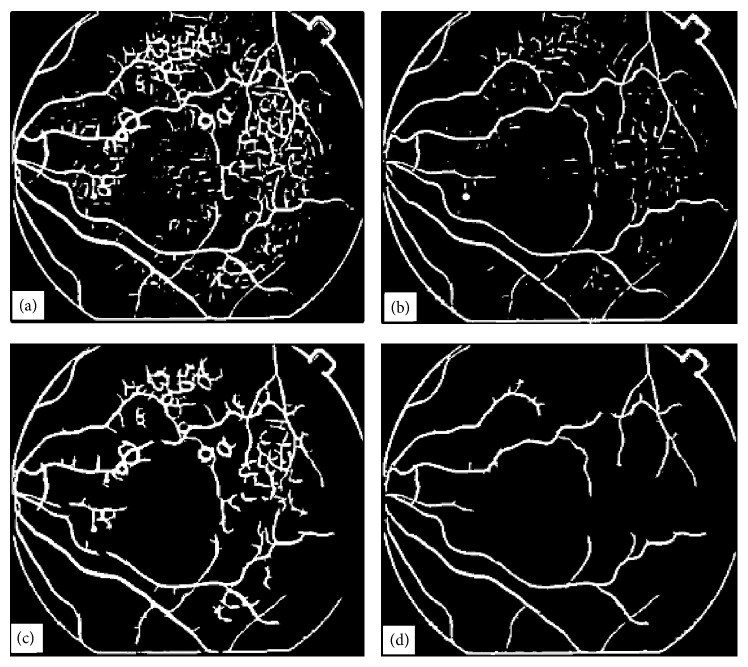
Comparison of vessel tree extraction with conventional matched and modified matched filtering techniques, (a) image obtained with conventional matched filtering approach, (b) image obtained with modified matched filtering technique (thresholded image obtained after processing with homomorphic filter and matched filter), (c) image obtained after length filtering of image in (a), (d) image obtained after length filtering of image in (b).

The method for the extraction of blood vessels is further modified to improve the quality of the extracted blood vessel tree. In this approach, the image background at corners is modified and then a homomorphic filter is applied to obtain smooth image. This will enhance the contrast of the image in comparison with the original. Since we are interested in enhancing the signal of the blood vessels which is lying in the range below the threshold value, the image is automatically thresholded using the OTSU algorithm keeping the information below the threshold value and giving the rest of the image the same value as the threshold. This has greatly enhanced the effect of the blood vessels.  Figure 14(a) shows the original image.  Figure 14(b) shows the same image with modified background.  Figure 14(c) shows the image obtained as a result of application of homomorphic filtering, and Figure 14(d) shows the image obtained by automatic thresholding of the image in Figure 14(c) by the OTSU method keeping the data of the image below the decided threshold unchanged and the data above the threshold being replaced by the threshold value. We have done this because our required data lies towards the lower end of the image data spectrum. This act has greatly enhanced the contrast of the image. Then this image is subjected to the matched filtering technique and thresholded automatically by OTSU. 100% correct results have been obtained for thresholding the image by this method, and there is no need to play around with the threshold value for better results as we needed to do with the conventional matched filtering method because now there is a big difference between background and signal; that is, between-class variance is increased and at the same time within-class variance has been decreased. The whole process is automated.  Figure 15(a) shows the result obtained with the conventional matched filtering method, and Figure 15(b) shows the result obtained with the modified matched filter method.

It has been observed that along with the enhancement of blood vessels in the abnormal images some other features are also sometimes enhanced and it is essential to eliminate them. The method used to filter them out is called length filtering. In length filtering a single identity is assigned to a single piece of connected objects and the number of pixels in each connected objects is determined and so they can be filtered easily. The effect of length filtering is shown in part (c) and (d) of Figure 15. We have tried to prevent information (other than blood vessels) being enhanced with the use of matched filters by making an effort to enhance the contrast of the blood vessels and at the same time to reduce the contrast of background features. In some cases, when the lines are small and disjoint, it is possible to filter them out completely but when they combine to form bigger structures then it is difficult to eliminate them. In Figure 15(b) the small lines in the image can easily be removed by the length filtering technique, but it is very difficult to clear the lines in the image in Figure 15(a) as they combine together to form structures with large numbers of pixels and any effort to clear those structures may lead to the loss of blood vessel information.  Figure 15(c) shows the image in Figure 15(a) after length filtering at 136 pixels, and Figure 15(d) shows the image in Figure 15(b) after length filtering at the same number of pixels.

**Figure 16:**
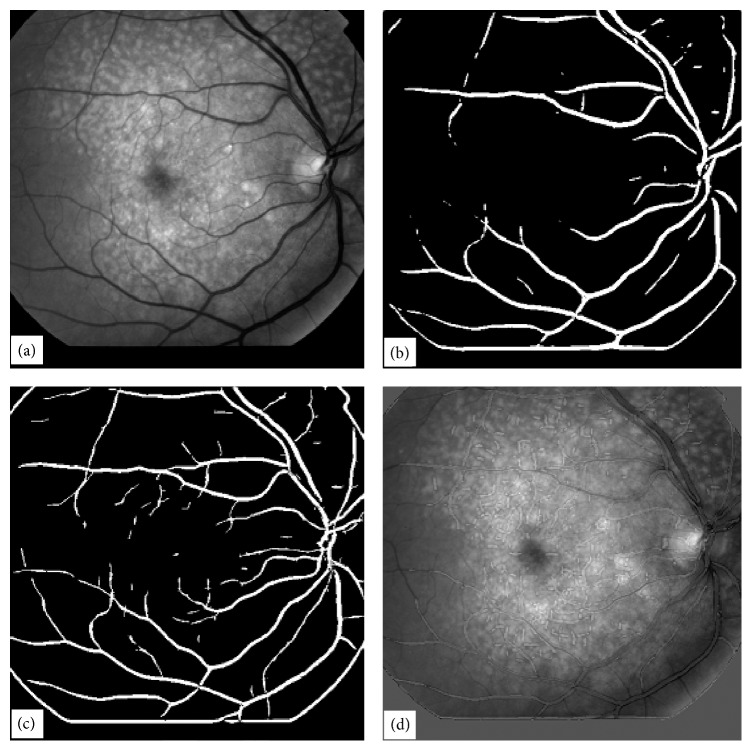
Results with modified matched filtering technique (a) original image, (b) image formed using one kernel, (c) image formed using 4 kernels of different lengths and widths, (d) image formed by overlapping the scene in (a) and the blood vessel tree in (c).

It has been found that if an image contains thin blood vessels, then by using a kernel of small width these blood vessels can be enhanced. One can try kernels of different widths, and a detailed tree of blood vessels can be extracted by combining the responses at each pixel from the images obtained by using kernels of varying widths. The results are shown in Figure 16. Figure 16(a) shows the original image. Figure 16(b) shows the blood vessel tree using single kernel of length 4 and width 10, and Figure 16(c) shows the blood vessel tree obtained using 4 different kernels of different widths and lengths. Figure 16(d) shows the tree extracted with kernel of length 2 and width 4 (without length filtering) overlapped with the unprocessed image. At certain points where the width of the blood vessels is more than the width measured by the matched filtering method, one can see black lines along the blood vessels, and where the width is less in the original scene than the width measured by the matched filter, one can see a black line in the central region with as outer region of low intensity which shows the enhanced width due to the matched filtering method. At the points where the matched filter correlates exactly with the width of vessels one can observe an exact overlap. It has also been observed that if the image is first processed with a high pass filter followed by matched filter method, then superior results are achieved. This is because the high pass filter can greatly enhance the edges of the vessels increasing the contrast of the vessels against background.

## 5. Blood Vessel Extraction (BVE) with Continuation Algorithm (CA)

One of the most frequently cited technique, for BVE is a matched filtering technique. This technique has its own drawbacks. The size of the kernel for matched filtering technique is very important. One kernel is not enough to extract the whole tree without significant noise. Moreover, extraction of thick and thin and intermediate sized vessels requires different sized kernels in the matched filtering technique. Different binary images obtained with different sized and different orientation kernels in the matched filtering technique are combined to give one binary vessel tree image, but in addition to this some noise filtering techniques are always required to filter out the segments which are not part of a vessel tree. The full automation of matched filtering technique requires careful selection of kernels of different sizes and orientations and of course a noise filtering technique as well. Even the best possible result with matched filtering technique has some drawbacks. One of the fundamental drawbacks is a discontinuity in the vessel tree especially where the vessels are very thin. This is because of the smaller signal to noise ratio for weaker or thinner vessels. Due to this discontinuity the application of any noise (broken segments of vessels) removal or length filtering technique would result in a vessel tree image which is not complete especially on the sides where thin vessels end. Sometimes thin vessels provide more important information to an ophthalmologist about the progression of a disease.

The other vessel extraction techniques as mentioned in [1–31] suffer from more or less similar defects especially when concerned with thin vessels. The broken thin vessels in the binary vessel tree if joined could give us a complete vessel tree, and then the other broken parts which are not the part of blood vessels can easily be removed using any noise filtering technique, for example, a length filtering technique. We felt the need to develop an algorithm that could join the broken parts of the vessel tree segments to develop continuity and at the same time identifying the noisy bits in the image for their removal if necessary.

In this section we present a novel technique for the development of continuity between the broken thin vessel segments in a binary vessel tree image avoiding the joining of broken segments which are not part of the actual vessel tree. In the proposed technique called Continuation Algorithm (CA) we generate two binary tree images of the given retinal image and process them to obtain a noise free continued segments vessel tree. The two binary images can be generated using matched filtering techniques [1, 10, 18, 19].

The proposed continuation algorithm requires two binary vessel trees from a given retinal image. We have used the matched filtering technique for the extraction of binary tree as it provides appropriate control to obtain two pre-requisite images for our proposed continuation algorithm. Other techniques for blood vessel extraction as cited in the reference list may be used provided two conditions are satisfied by the binary tree images: (1) the first binary image (refer to it as reference binary image) should contain thick blood vessel tree structure which is obtained with matched kernel of relatively larger size. The reference image would contain only thick vessels and no noise, that is, no broken segments. If there are any broken segments length filtering or any other noise filtering technique could be used. (2) The second binary image (refer to it as test image or image2) should contain thick as well as thin vessel tree structure as it is obtained with matched filtering the retinal image with a smaller kernel size. As the image2 contains thin vessels, it also contains a lot of noise.

**Figure 17:**
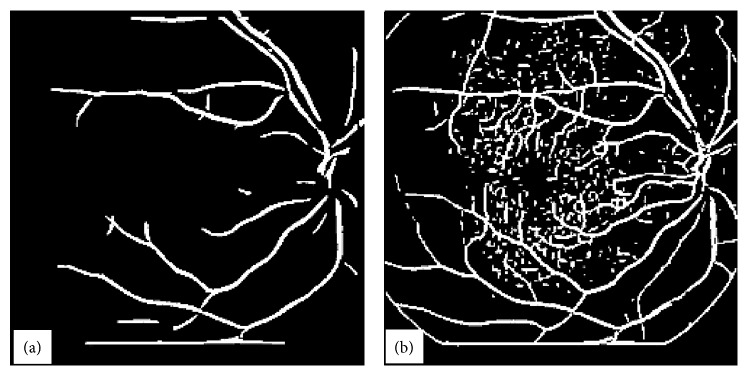
Display of two binary images needed for Continuation Algorithm, (a) reference image obtained with matched filtering technique and thresholded with OTSU, (b) second binary vessel tree image obtained with smaller matched filtering kernel and thresholded with OTSU.

A typical low quality abnormal retinal image chosen for simulation using CA is shown in Figure 1(a). The image chosen for the simulation is abnormal in the sense that it consists of drusen, it is a poor quality image in terms of contrast, the contrast of thin vessels is extremely poor, and it is hard to visualise the ending points of thin vessels being submerged in the background noise. Two binary vessel trees are extracted from a retinal image using the matched filter technique. One binary tree image called the reference image consists of a thick vessel tree and can be made free of noise by the application of a length-filtering algorithm if required. The reference image is shown in Figure 17(a). The second binary tree image (called image2) required by the algorithm consists of thick as well as thin vessels. A lot of noise appears in the image as a result of the application of matched filtering. This image is shown in Figure 17(b). The noise in this image cannot be filtered out using a length-filtering technique as most of the thin vessel structures will also be filtered out. Matched filters with different kernel sizes and with different orientations were used to obtain the two binary vessel trees. The main drawback of the matched filtering technique is that it is poor at extracting noise-free thin vessels.

The binary reference image is multiplied by a large integer, say 100. The pixel values in the image are either 0 or 100. For each pixel (*i*, *j*) in the reference image where the value is zero that is, refimg (*i*, *j*) = 0, and for the same pixel (*i*, *j*) in the second binary image where the value is 1, that is, img2 (*i*, *j*) = 1, a sum of pixel values is calculated as

(14) sum=refimg(i,j+1)+refimg(i+1,j+2)+refimg(i+1,j+3)+refimg(i+1,j+1)+refimg(i+2,j+3)+refimg(i+2,j+1)+refimg(i+3,j+2)+refimg(i+3,j+1)+refimg(i+1,j).

**Figure 18:**
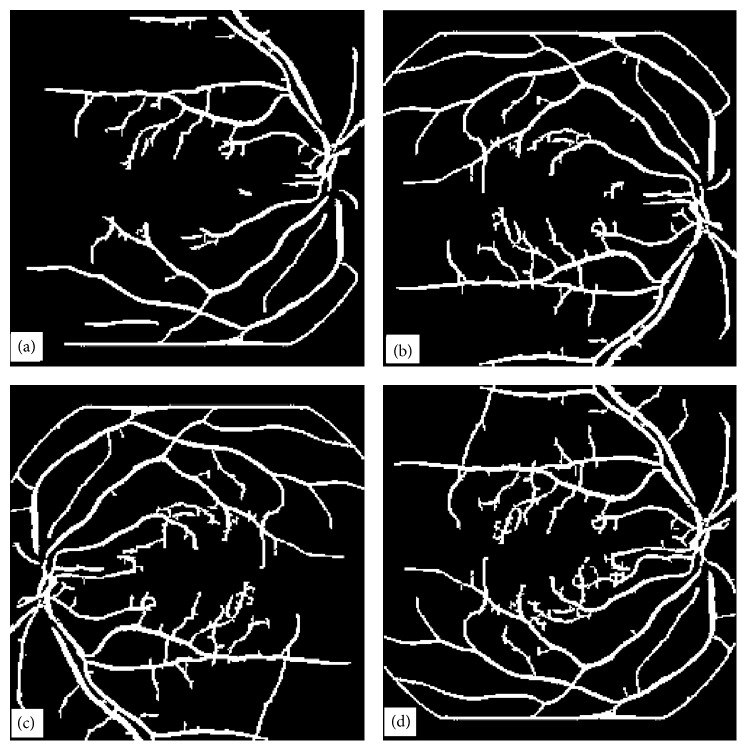
Application of Continuation Algorithm to a typical poor quality retinal image, (a) image obtained from the image in Figure 17(a) in the first pass of continuation algorithm, (b) image obtained in the second pass of continuation algorithm applied on the vertically flipped version of the image shown in (a), (c) image obtained in the third pass of continuation algorithm applied on the horizontally flipped version of the image shown in (b), (d) image obtained in the fourth pass of continuation algorithm applied on the vertically flipped image in (c) and shown after horizontal flip.

If the sum ≥ 100, then the pixel value in the reference image is set to 100, that is, refimg (*i*, *j*) = 100. This process is repeated indefinitely unless all the zero value pixels in the refimg find no unity value pixel in the img2 for all (*i*, *j*) in the refimg. The algorithm works in four steps. First it extends the tree in the reference image from top to bottom using the information from the second binary image. The tree extension obtained in the first step is shown in Figure 18(a). The vertically flipped version of the image shown in Figure 18(a) is then passed to the algorithm. The image after the second application of the algorithm is shown in Figure 18(b). The image in Figure 18(b) is then flipped horizontally and the algorithm is applied. The resultant image is shown in Figure 18(c). The image in Figure 18(c) is then flipped vertically and after the fourth application of the algorithm the resulting image is filliped horizontally and is shown in Figure 18(d). The comparison of the two images (image in Figure 17(b) and image in Figure 18(d)) reveals that our proposed continuation algorithm gives reasonably accurate vessel tree structure of a poor quality retinal image. The algorithm has been tested on 30 images for vessel tree extraction and the performance has been found to be satisfactory.

## 6. Discussion and Comparison of Proposed Vessel Extraction Techniques

**Figure 19:**
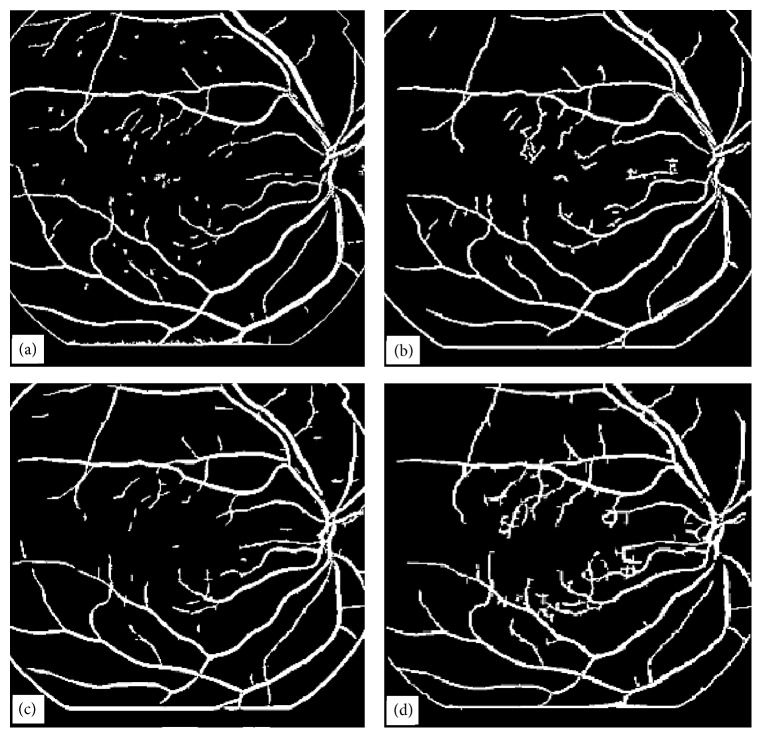
Display of blood vessel trees obtained with four different techniques, (a) vessel tree image obtained using ILCS technique, (b) vessel tree image obtained using EEED technique, (c) vessel tree image obtained using MMF technique, (d) vessel tree image obtained using CA.

In this paper, we have presented four vessel tree extraction techniques. Each technique was tested on the same set of 30 highly abnormal poor quality retinal images. The four techniques have also been evaluated on 105 normal retinal images. Most of the techniques in the literature can work well on normal images but fail to perform well on abnormal retinal images. In this paper, we have focussed mainly on the abnormal retinal images. A set of four typical abnormal images are shown in Figures 1(a), 4(a), 9(a), and 14(a). The image in Figure 1(a) was processed by all the four techniques and their results have been reproduced for comparison in Figure 19. Images in Figures 1(a) and 4(a) were processed with ILCS, image in Figure 1(a) was processed by EEED, images in Figures 1(a), 9(a), and 14(a) were processed by MMF technique and the image in Figure 1(a) was processed with CA. We have chosen to present the image in Figure 1(a) as a typical abnormal retinal image for comparison.

The four techniques in the order of increased simplicity are MMF, ILCS, EEED, and CA. MMF being relatively more complex has been discussed in greater detail and three retinal images have been presented to support the technique. ILCS which is comparatively less complex has been demonstrated with two retinal images and the rest of the two techniques EEED and CA being much simpler have been supported by one image each. The images obtained with EEED and CA shown, respectively, in Figures 19(b) and 19(d) are superior in quality to the images obtained with ILCS and MMF shown, respectively, in Figures 19(a) and 19(c). The vessel tree structure obtained with ILCS (Figure 19(a)) contains more isolated segments or broken vessels whereas the broken vessels in the image Figure 19(c) by MMF are relatively less. The image in Figure 19(b) by EEED contains a few broken pieces of vessels and the image in Figure 19(d) by CA contains almost no broken vessel part. This is the beauty of CA method.

The MMF technique was found to be computationally more expensive. The four techniques (ILCS, EEED, MMF, and CA) were run for the image in Figure 1(a) on the machine: Pentium(R) D CPU 3.4 GHz, 2 GB of RAM, Microsoft Windows XP Professional Service Pack 2 Version 2002 and Mathematica 4.1 in a stand-alone mode. The measured computational time was 1 min. 14 seconds 200 milliseconds for ILCS, 1 min. 1 second 99 milliseconds for EEED, 1 min. 49 seconds 460 milliseconds for MMF, and 40 seconds 69 milliseconds for CA. The four techniques can be arranged in the order of increasing time consumption as CA, EEED, ILCS, and MMF. For rest of the 29 images the trend was maintained but with small relative differences in measured time values.

The four images shown in Figure 19 were presented to three expert human graders for evaluation purposes. Their averaged ratings for the images in Figures 19(a), 19(b), 19(c) and 19(d), respectively, were 76%, 88%, 82%, and 97%. For the rest of the images, CA was always evaluated in the range (95 to 99%), EEED was always evaluated in the range (88 to 91%), MMF was always evaluated in the range (80 to 85%), and ILCS was evaluated in the range (74 to 78%). These results were found when the relevant codes were run in fully automatic mode requiring no human intervention during the processing time. It has also been observed that the techniques ILCS and MMF can be improved by 1 to 2% if human intervention is involved for some images. The techniques CA and EEED were found to be fully automatic and even a human intervention could not improve the results obtained with them. The four techniques in the order of improved automation may be listed as MMF, ILCS, EEED, and CA. These techniques when analysed on normal retinal images gave improved results: (99% to 100%) for CA, (96% to 98%) for EEED, (94% to 96%) for MMF, and (88% to 93%) for ILCS. It is worth noticing that even the results of the expert human graders varied by 2.5% and we averaged their results for comparison with our results.

Most of the techniques mentioned in [1–31] extract binary vessel trees of retinal images embedded in noise. Such techniques extract the vessel trees along with noise like the one shown in Figure 17(b). Some types of noise filters are then used to remove noise but at the cost of losing parts of the vessels which are very thin. The reason is that the very thin parts of the vessel trees are usually treated as discontinued segments by the existing noise removal techniques. The trade-off in removing the noise is to lose parts of the vessel trees which are very thin and the resulting vessel tree structures do not represent the true picture. The existing vessel tree extraction techniques either give up the extraction of thinnest part of vessel trees like the image shown in Figure 17(a) or produce images with a lot of noise as shown in Figure 17(b). The proposed continuation algorithm presents a reasonable solution to these problems. It retrieves noise free vessel tree images and at the same time preserves the thinner vessel segments in the vessel trees.

Some of the existing vessel tree extraction techniques [1–32] produce reasonable tree images for only those retinal images which are good in quality, in contrast and in appearance and in which the vessel tree can be perceived easily with naked eye. The existing techniques do not seem to give reasonable solution when applied to the poor quality images like the ones as shown in Figure 1. We deliberately chose an image with poor quality, with poor contrast, and with poor appearance in which the thinner parts of the vessel tree structure are hardly visible with naked eye. The image (Figure 1(a)) also contains a lot of drusen which act as embedded noise. In the presence of these abnormalities in the processed image, the proposed method based on continuation algorithm seems to give accurate results in the extraction of full tree structure retaining the thinner vessel parts and at the same time removing the noise as its inherent capability. The technique does not require the application of any noise removal technique during its processing steps if the matched filter is carefully chosen in obtaining the reference image used in the proposed technique.

The proposed continuation algorithm ensures continuity in the extracted vessel trees till the end of the thinner parts of the vessel segments. This is the first main characteristic of the CA technique. The existing vessel extraction techniques find it hard to ensure this characteristic and a discontinuity is observed especially with poor quality and noisy images. The built-in feature of the continuation algorithm is such that it removes the noise during its processing steps and does not require any postprocessing which is its second main characteristic. This type of characteristic is lacking in existing techniques and some existing techniques may add noise in the vessel tree images during processing and require some postprocessing for noise removal. If retinal images are available from the same Fundus camera, the matched filtering kernel used in obtaining the reference image will be fixed and the continuation algorithm can work without any human intervention. This will also exempt the user from any sort of pre-processing. This feature is also unique to the proposed CA technique. That is, CA does not require any pre- or postprocessing in the sense required by other vessel extraction techniques.

## 7. Conclusion

Four different methods for BVE have been reported in this paper. Filtering of noise (removal of nonvessel segments) is an essential part of many BVE methods for clean outputs. The CA method does not require any sort of filtering at any stage. The other three methods (ILCS, EEED, MMF) and most of the methods given in the references require some sort of filtering to remove the broken vessel pieces, pieces of leftovers of other removed segments which were not vessels and the background noise.

We have mentioned the success rate for each of the four techniques. If the success rate for a given technique is, for example, 95%, then it means that only 5 pixels out of 100 pixels differed from the averaged ground truth image constructed by the three expert human graders. The success rate of 95% does not mean that a particular technique worked only for 95 images out of 100. All the four techniques worked for all the test images including 30 abnormal images and 105 normal retinal images but with different accuracies. Results of the three expert human graders in the form of ground truth images were found to vary by a maximum of 2.5%. The interpretation of our success rate is different from the meaning of the success rate used in the literature.

Two noise removal techniques were developed to remove noise from the final binary vessel trees. One was the length-filtering technique and the other was a noise removal algorithm. Results have been presented which indicate the performance of these noise removal techniques. When employing a length filter technique a threshold value is passed to the algorithm. Nonvessel structures having pixel values less than the threshold value are deleted. The appropriate value for the threshold has been found to vary from 10 to 50 depending upon the noise level in the input retinal image. From automation point of view a value of 50 for the threshold gives good results. In the length filtering algorithm the isolated structures in the binary vessel tree are grouped and the number of pixels in each group is computed. Groups with number of pixels smaller than a threshold are removed. The noise removal algorithm works in an entirely different way. A square window of size (*x*, *x*) pixels is centred at each pixel position in the binary vessel tree. If all the boundary pixels are zero, then the data within the window is all set to zero. The appropriate window sizes vary from (8, 8) to (16, 16). This algorithm was found to give satisfactory results, and the validity has been established on 30 abnormal retinal images and 105 normal retinal images. The noise removal algorithm is computationally less expensive than the length-filtering algorithm.

The most commonly discussed method in the literature is matched filter method. We have suggested a modification to the existing method that gives better results as compared with the results obtained using the ordinary or conventional matched filtering technique. Our method of matched filtering includes some preprocessing of the retinal images. In the ordinary or conventional method, a matched filter is directly convolved with a retinal image, which also enhances nonvessel structure in the retinal image and gives rise to noise in the binary vessel tree structure. In our method, homomorphic filtering is first applied to the retinal image. A threshold value is computed for the image using the OTSU threshold method and all the data in the image greater than the threshold is replaced by the threshold value. The resulting image may have uniform background illumination with vessel structure more enhanced as compared with the preprocessed retinal image. A matched filtering technique is then applied to extract the vessel tree structure. A threshold value is then computed using the OTSU method and a binary vessel tree is produced with reduced nonvessel structure in the image. The modifications we have suggested have greatly improved the vessel tree structure as compared with that obtained using only the conventional matched filter technique. Drawbacks of using only one matched filter to recover the vessel tree have been discussed. We have also introduced a multimatched filtering technique in which a preprocessed retinal image is convolved with matched filters of different kernel sizes and corresponding binary vessel tree images are combined at the end. The resulting image has thick and thin vessels recovered precisely. The results reveal that the modified matched filtering technique could be a promising technique for BVE in retinal images. Kernels of different lengths and widths with all possible orientations (with a minimum step of 9°) should act as matched filters for a given retinal image. The matched filtered images are then combined to construct vessel tree structures. Preprocessing of retinal images and post processing of uncleaned binary trees are the important ingredients of vessel tree extraction process by MMF method.

In the continuation method two binary vessel trees using matched filtering techniques are used. One of the binary vessel tree images contains only thick vessels and is noise free which we named as the reference image. The second binary vessel tree image contains thick vessels, thin vessels, and a lot of noise. The two binary images are used in the developed algorithm. The proposed method extends the vessel tree in the reference binary image with the help of the other binary vessel tree structure. This process is repeated four times for different orientation of the images generated from the reference image with the application of continuation method. This process extends the vessel tree in the reference image according to the vessel tree in the second binary image except where the nonvessel structure (noise) occurs. The final vessel tree obtained from the reference image with successive applications of the continuation algorithm is an exact copy of the second binary vessel tree image except that the nonvessel tree structure is absent. This image has thick and thin vessel tree structures and is noise free. This technique does not require noise filtering at any stage as required by other vessel tree extraction methods. Most noise filtering techniques also filter out some parts of the vessel tree. We have tested our continuation method on 30 poor quality abnormal retinal images and on 105 normal (good quality) retinal images and found the technique to work satisfactorily. The success rate of the CA method was 95% to 99% when tested over 30 abnormal retinal images which approach to 99-100% for normal retinal when tested over 105 normal images. The technique is superior to existing vessel tree extraction techniques and the other three techniques (ILCS, EEED, and MMF).

We conclude that the four techniques for vessel tree extraction from retinal images are good additions to the existing vessel segmentation techniques and the two techniques: EEED and CA, are especially important as they satisfied the human graders who recommended these two techniques for BVE from retinal images on commercial grounds.
